# Exploring views and experiences of a unique alcohol assertive outreach model, the primary care alcohol nurse outreach service (PCANOS): a qualitative study

**DOI:** 10.1186/s12875-025-02755-8

**Published:** 2025-03-03

**Authors:** Clare Sharp, Andrea Mohan, Danielle Mitchell, Niamh Fitzgerald

**Affiliations:** 1 Institute for Social Marketing and Health, University of Stirling, Stirling, UK; 2 School of Health Sciences, University of Dundee, Dundee, UK; 3 Usher Institute, University of Edinburgh, Edinburgh, UK

**Keywords:** Alcohol, Alcohol nurse, Service engagement, Assertive outreach

## Abstract

**Background:**

There are recognised barriers to engagement with mainstream alcohol services for certain groups within populations. Alcohol assertive outreach is an approach that uses repeated, persistent and flexible methods to engage with patients with alcohol problems from these groups. There are few qualitative studies that explore how alcohol assertive outreach services are experienced by stakeholders. This study focuses on a unique service, The Primary Care Alcohol Nurse Outreach Service (PCANOS), that operated in Glasgow, Scotland and which involved Alcohol Nurses working closely with general practices.

**Methods:**

Twenty-three semi-structured qualitative interviews were used to explore staff and patient views and experiences of PCANOS. Interviews were conducted with 18 staff (nine general practice staff, five alcohol nurses, and four strategic staff) and seven patients from across six Deep End general practices.

**Results:**

Findings from this study suggest that PCANOS has the potential to engage patients who may have difficulties engaging with mainstream alcohol treatment services. Through PCANOS, the Alcohol Nurses, in collaboration with General Practitioners and other practice staff, were able to engage patients and deliver a flexible, person-centred care service that impacted positively on patients’ drinking behaviour and general health and wellbeing.

**Conclusions:**

PCANOS was a unique alcohol assertive outreach service that had the potential to engage with people from the most deprived communities in Glasgow, who were not engaging with the mainstream services. Further research could examine the potential benefits of services like PCANOS, including patient outcomes, the economic impact on the wider healthcare system, and its transferability to other settings such as rural areas.

**Supplementary Information:**

The online version contains supplementary material available at 10.1186/s12875-025-02755-8.

## Introduction

Alcohol causes significant harm to health, wellbeing and the economy. In Scotland, alcohol-specific deaths have generally been rising over the past decade; in 2023, 1,277 people died from causes specifically attributed to alcohol, the highest recorded since 2012 [1]. It is estimated that alcohol harm costs the Scottish society and economy between £4.9 to £9.6 billion [2]. People from the most socioeconomically deprived areas in Scotland are the most impacted by alcohol-related harm. They are 4.5 times as likely to die from alcohol-specific causes [1] and eight times as likely to be hospitalised because of alcohol-related causes, compared to people from the least deprived areas [3]. They are also more likely to suffer from severe multiple disadvantages including homelessness and poor mental health, and are less likely to engage with health and social care services [4, 5].

In the UK, support for people with alcohol problems is provided through primary and secondary care. In this paper, we use ‘alcohol problems’ as an umbrella term to refer to the range of conditions relating to alcohol use that is harmful or potentially harmful to an individual’s health including diagnosable Alcohol Use Disorders (AUD) (which include alcohol dependence) [6]. Primary care is delivered by a range of healthcare professionals who serve as the first point of contact for patients. These professionals include those working in general practice teams such as general practitioners (GPs) and practice nurses; community nurses; and dental professionals. General practices are ideal settings for managing alcohol problems as they are located in communities, are accessed by a large percentage of the population, and are likely to encounter people with alcohol problems. GPs can have supportive conversations to help patients understand and consider the impact of, and potentially reduce, their drinking [7, 8]. They can also refer patients who require more specialist support on to appropriate services where available [9]. Secondary care settings such as hospitals and alcohol services based in the community, can also support people to manage their alcohol problems [10].

There are several challenges to supporting alcohol problems in primary care. GPs and other healthcare professionals usually have time limits for patient appointments, or may lack the necessary skills or resources to adequately support people with alcohol problems [11–14].There are also challenges for secondary care services that provide alcohol support including budget cuts, competing priorities (for example, in the case of combined alcohol and drugs services), and a lack of patient engagement due to factors including the stigma associated with attending an addiction service [15–19].

Assertive outreach services aim to build relationships with and support people who do not engage with mainstream services including alcohol support and mental health services [20]. While there is no one definition of assertive outreach, there is consensus that it involves several key components including persistent and repeated attempts to contact people, taking services outside of traditional settings to where target populations are located (for example, in their homes), and provides flexibility and holistic care [20]. There is limited research about assertive outreach that focuses on alcohol problems, however, evidence from a few evaluations shows that when provided by multi-agency teams, alcohol assertive outreach can help people with AUD by improving their engagement with mainstream services and reducing their alcohol use [21–24]. Different models of alcohol assertive outreach exist, however, there are few qualitative studies that explore how these are experienced by the people who deliver the services, and by the people who use them. Understanding these experiences can contribute to a more in-depth understanding of how these models work in practice.

This study focused on a unique model of alcohol assertive outreach that operated in Glasgow, Scotland from 2015 to 2016 and 2019–2024. The Attached Alcohol Nurse (AAN) initiative (secondary care service) which ran between 2015 and 2016, involved two full-time specialist addiction nurses (referred to as Alcohol Nurses (ANs) in this paper), that worked closely with seven Deep End general practices (primary care service) [25]. Deep End general practices are those serving the 100 most deprived communities in Scotland [26, 27]. The ANs supported GPs by providing specialist alcohol treatment and other support to patients of the Deep End practices who were referred to the AAN initiative. The pilot was funded by the Glasgow City Alcohol and Drug Partnership (ADP) and following a report of the successful partnership working between GPs and the ANs, the AAN was implemented for a longer period [25]. It was renamed the Primary Care Alcohol Nurse Outreach Service (PCANOS), which ran from 2019 to 2024. Our study aimed to explore the views and experiences of stakeholders of PCANOS and how it compared to mainstream alcohol services in Glasgow. This paper builds on findings published in a report for the funder of the study in 2022 [28].

## Methods

### Design

This was an exploratory qualitative study that used semi-structured interviews to collect data to analyse the views and experiences of PCANOS as an alcohol assertive outreach service and how it compared to mainstream alcohol services provided in Glasgow. Ethical approval to conduct the study was gained through the East of Scotland Research Ethics Service (19/ES/0127) in November 2019. A Study Steering Group was set up to provide guidance to the research team and support the running of the study.

### Sampling and recruitment

We recruited a range of stakeholders who could provide different perspectives of PCANOS including Deep End practice staff, strategic staff (individuals who were involved either in the development or rollout of the AAN initiative and PCANOS, or who had a strategic role in managing alcohol problems in Scotland), ANs and patients who used PCANOS.

A list of 10 Deep End practices that had signed up to refer eligible patients to PCANOS (*n* = 10) was obtained via the lead AN of PCANOS; of these practices, six had taken part in the AAN initiative in 2015–2016, while four had signed up to PCANOS after the service recommenced in 2019. We approached all 10 practices and six agreed to participate (four of these had taken part in the AAN initiative). Practice staff from all six practices were recruited and included GPs, Practice Managers, and Link Workers (specially trained staff who support patients with complex health and social care issues) (Table 1).

**Table 1:** Practices and staff

Deep End GP practice	Took part in AAN or PCANOS	Participants
Practice A	AAN & PCANOS	1 GP
Practice B	AAN & PCANOS	1 GP
Practice C	AAN & PCANOS	1 GP
Practice E	AAN & PCANOS	1 PM 1 LW
Practice I	PCANOS	3 GPs
Practice J	PCANOS	1 LW
Total no. participants from GP practices		9

^GP– General Practitioner; PM– Practice Manager; LW– Link Worker^

We obtained a list of strategic staff via the Study Steering Group. Six individuals were identified and all contacted, and of these, four agreed to participate. We contacted all the five ANs who were employed with PCANOS at the time of the study and all agreed to participate. The ANs passed on information about the study to patients who were referred to, or were engaging with PCANOS at the time of the study. Patients who were interested in participating consented for the ANs to give their contact details to the research team. In total, 14 patients were contacted and seven agreed to participate. In total, we conducted 23 qualitative semi-structured interviews with 25 participants (two were paired interviews with two ANs) (Table 2).

**Table 2:** Participant characteristics

Interview No.	Role	PCANOS status	Gender
1	Addiction Nurse	-	F
2	Addiction Nurse	-	F
2	Addiction Nurse	-	M
3	GP	-	F
4	Strategic staff (Analyst)	-	F
5	GP	-	M
6	Strategic staff (Nurse)	-	F
7	Strategic staff (Alcohol Treatment Manager)	-	M
8	Practice Manager	-	F
9	Link Worker	-	F
10	GP	-	M
11	Link Worker	-	F
12	Strategic staff (GP)	-	F
13	GP	-	M
14	GP	-	M
15	GP	-	M
16	Patient	Active	M
17	Patient	Discharged	M
18	Patient	Active	M
19	Patient	Active	F
20	Addiction Nurse	-	F
20	Addiction Nurse	-	M
21	Patient	Discharged	M
22	Patient	Discharged	F
23	Patient	Active	M

### Data collection

Two researchers conducted the interviews between September 2020 and June 2021 using telephone or Microsoft Teams. The COVID-19 pandemic occurred during this period but PCANOS still provided support to patients. We developed four topic guides with input from the Study Steering Group (see files in Supplementary Information). They were tailored to each of the participant groups (alcohol nurses, frontline practice staff, strategic staff and patients) but covered some broad themes including participant background, experiences and views of PCANOS, and experiences and views about mainstream community-based alcohol services.

### Data handling and analysis

Interviews were recorded, transcribed professionally by an external agency and transcripts checked by the research team for accuracy before beginning analysis. All transcripts were anonymised prior to analysis to remove any identifiable information. We used a thematic analysis approach following the six-stage approach outlined by Braun and Clark [29, 30]. Three researchers independently reviewed a sample of the transcripts representative of different participant groups to ensure familiarity with the data and used a deductive (informed by the topic guide) and inductive (allowing for additional codes identified in the data) approach to identify initial codes. The researchers then met to discuss and agree a final coding framework which was subsequently applied when coding all of the transcripts in NVIVO. A summary table of the coding framework is available in Supplementary Information. The research team met regularly to discuss and resolve any coding queries throughout this process. Once NVIVO coding was complete, the researchers met to review the coded data and identify and name the themes emerging from the data. The findings are presented under these themes in the form of data summaries and selected quotes to illustrative these.

## Results

Seven themes were identified from the data analysis: (1) a person-centred approach to care, (2) collaborative working, (3) a co-ordinated approach to care, (4) support from families, (5) stigma and trauma relating to alcohol problems, (6) co-existing mental health or other addiction problems, and (7) impact of the COVID-19 pandemic. We first present a description of the PCANOS model below, before elaborating on the seven themes, and presenting a summary of the comparison between PCANOS and other mainstream community services.

### The PCANOS model

PCANOS aimed to engage with patients from Deep End practices whose alcohol consumption was already negatively impacting on their health and who were not engaging with the mainstream Alcohol and Drug Recovery Services (ADRS) in Glasgow. Overall, the patients’ age and employment status were varied, but most patients tended to be men and living in areas where there was a high level of deprivation. Most patients were referred to PCANOS by staff from their general practice, mainly the GPs, but some referrals were also made by the mainstream alcohol services and the Accident & Emergency services. Once the PCANOS team received a referral, they aimed to contact the patient via telephone within 24 hours. ANs also made repeated attempts to contact referred patients beyond this point, until the patient responded. During the initial telephone contact, ANs would arrange to visit the patient at their home or at their general practice at the patient’s earliest convenience.

At the first visit, the ANs would complete a comprehensive health and wellbeing check with the patient, and discuss what patients wished to achieve from engaging with the service. ANs would then agree on a tailored treatment plan with patients. The ANs adopted a harm reduction approach to patients’ alcohol consumption, whereby they aimed to support patients to reduce their consumption, rather than requiring total abstinence. The ANs were skilled to provide a range of interventions including home-supported alcohol detoxification, advice on diet and sleep, and psychosocial interventions. They also referred patients, and guided them where necessary, to other community support. The ANs worked closely with GPs to support patients by providing regular updates of the patients’ progress and reviewing their treatment plan, including any medication, on a frequent basis. The ANs supported patients for 13 weeks, during which time they sought to build trust and give them the confidence to engage with the mainstream services before discharging them from PCANOS.

### Theme 1: a person-centred approach to care

A core element of PCANOS that was consistently described by participants was the use of a person-centred approach that helped engagement with patients– *“Meeting the patient’s individual needs*,* it’s all about patient-centred*,* individualised care.”* (Interview 12, Strategic staff). This approach was appreciated by patients as it helped to foster trust with the ANs– *“.it was a lot more personal. As I say*,* she* [AN] *was taking an interest in me.”* (Interview 17, Patient). A key component of the person-centred approach was providing flexibility when scheduling appointments with patients, for example, arranging visits at the patients’ homes where they were most comfortable, making use of alternative contact methods during the COVID-19 pandemic such as video calls, and scheduling visits with patients who worked outside of their working hours:

*“… that’s about people being able to engage in the service* [PCANOS] *because sometimes with the addiction services*,* it is 9am to 5pm*,* so time is restricted so people don’t get access to*,* if they want assessed or whatever*,* so it’s about being flexible and giving the person choice and access to the service as well.” (Interview 2*,* Alcohol Nurse)*.

Another key component of the person-centred approach was the ANs’ abilities to provide tailored treatment plans and knowledge of how to motivate individual patients. This aspect reportedly helped patients to improve different aspects of their health or wellbeing, such as reducing their alcohol consumption, or improving their diet, sleep or mental health:

*“The first couple of weeks was kind of hard for me*,* because I was drinking a litre a day basically*,* Smirnoff. I got it down slowly and gradually and every week she* [AN] *was coming in and she said you’re doing great. I’m eating now as well and I’ve had a jag* [injection] *as well*,* to get my appetite back up again.” (Interview 16*,* Patient)*.

*“She* [AN] *comes in and she just…it’s only been 2 months*,* 3 months but I feel so relaxed and so…even when I speak to her on the phone I go…I breathe I don’t feel anxious.” (Interview 19*,* Patient)*.

### Theme 2: collaborative working

Collaborative working between the PCANOS team and the general practice staff was another key element of PCANOS, and was perceived by the ANs, general practice staff and some strategic staff as beneficial to enhancing patient care and support, even for patients not referred to PCANOS. For example, Link Workers were able to draw on the ANs’ expertise when supporting patients with an alcohol problem who were also experiencing social issues.

The ANs had a base within general practices which allowed them to become more familiar with the practice staff, establish better communication in relation to patient care, and raise awareness of PCANOS across the practice team. This ultimately helped practice staff to feel supported by the ANs and led to more referrals to PCANOS.

*“Nurses were physically present in the health centre at least once a week so you were able to have a chat with them “oh I wasn’t sure what to do with this person*,* what do you think?” type conversation*,* so that kind of informal as well as formal referral process has been hugely important.” (Interview 10*,* GP)*.

*“It’s lovely when a GP practice is really motivated and enthusiastic about the service and they want to meet you and they are coming into us about it. This is the offer and this is what we are doing and we just start referring onto you*,* so it’s great when that happens.” (Interview 20*,* Alcohol Nurse)*.

However, while some general practices were supportive of the ANs and of PCANOS, there were some that weren’t. ANs perceived that this lack of support was due to many reasons, for example, that GPs were not accustomed to this ‘new’ approach to working, or that GPs had their own beliefs regarding how to support patients with alcohol problems. Ultimately, the lack of GP support was perceived as a barrier to referrals to PCANOS:

*“The service wasn’t embraced by them at all and I think because the GPs didn’t embrace it*,* they didn’t get the rest of the staff onboard.” (Interview 1*,* Alcohol Nurse)*.

### Theme 3: a co-ordinated approach to care

By working collaboratively, the PCANOS team was able to establish good communication with general practice staff, and were able to share patient information and discuss patient care efficiently. This led to another core element of PCANOS, which is the use of a co-ordinated approach to patient care. This included linking patients to other appropriate services such as the Link Worker service, hospital services, and mental health services. By doing this, the ANs helped patients to experience an easier and quicker transition into these services:

*“…recently I had a man who needed a detox*,* but we couldn’t detox him at home because he lived alone*,* he had previous self-harming behaviour*,* overdosing and things*,* but I managed to engage him to the extent that he had*,* where he had agreed to go into one of the in-patient wards*,* so I had done all that work with him*,* so then when I referred him into the* [Community Addiction Team (CAT)] *team*,* he was already on the waiting list so we had by-passed quite a few months……he was handed over to a worker within the CAT team*,* but all of that initial ground work was done by us.” (Interview 1*,* Alcohol Nurse)*.

### Theme 4: support from families

The ANs and patients’ accounts highlighted another key component of PCANOS, the use of family support to help motivate patients to achieve their goals. Wherever possible, the ANs tried to encourage the patient’s family to be involved in the patient’s daily journey to reduce their alcohol consumption, and to encourage patients to share more of their challenges with their families. Patients whose families had become involved reported that their family’s support was useful and appreciated, particularly during difficult times. A few patients also reported that since engaging with PCANOS, their relationship with their family members had improved:

*“I’ve got a wee chart*,* I’ve got a spreadsheet that my daughter done for me. I take what I have*,* one unit*,* one bottle of beer or whatever it may be. My wife marks it down*,* what my mood is*,* what food I’ve taken etc.*,* on a daily basis… It’s usually my wife or my daughter is in when she* [AN] *comes. They talk to her* [AN] *as well*,* which helps me as well*,* because if I tell her any tales*,* they’re going to say no*,* you done this or you done that… Again without the family - they have been very supportive since the first big blowout I had.” (Interview 16*,* Patient)*.

### Theme 5: stigma and trauma relating to alcohol problems

While there were several factors that supported engagement with the PCANOS team and with healthcare professionals in general, there were others that made this engagement a little more difficult. Two such factors were the self-conceptualised stigma that patients had in relation to their own alcohol problems, and trauma that they carried from past experiences. Both factors were identified in patient and AN interviews, and impacted on the ability of patients to disclose the truth to, or to quickly trust the ANs:

*“I went through abuse when I was young. But you don’t*,* that’s not a normal conversation you have with people. You have that with somebody you really trust….” (Interview 18*,* Patient)*.

*“I had one young guy who*,* he was stabbed when he was seventeen… his alcohol and mental health problems all stemmed from that young age you know… And for him to speak about this trauma*,* also his dad died of cancer… the hospital said his dad was going to be fine and then he ended up with a brain tumour*,* so for the NHS services that he’s been involved with*,* he can’t have trust. You know. So I had to build up the relationship.” (Interview 20*,* Alcohol Nurse)*.

### Theme 6: co-existing mental health or other addiction problems

Another factor which challenged the ANs’ abilities to address patients’ needs was the co-existence of mental health and/or other addiction problems alongside the patients’ alcohol problems. The ANs spoke of the conflict and complexity around working with other services to effectively support these patients:

*“So sometimes addiction and mental health get into this sort of a fight - no it’s your responsibility*,* no it’s yours. So*,* if we can get that person sober and abstinent then we can say well now you need to look at their mental health*,* you know?” (Interview 2*,* Alcohol Nurse)*.

### Theme 7: impact of COVID-19 pandemic

As this study was conducted during the COVID-19 pandemic, this event was referred to in the interviews with the GPs, ANs, and patients.

The GPs reported the impact of reduced frequency of meetings with the PCANOS team during the pandemic, though both teams still made efforts to communicate via telephone or email:

*“… unfortunately with COVID we haven’t been able to see him* [AN] *and his team face to face much*,* but previously the Nurses were physically present in the health centre at least once a week… It has been replaced to an extent with email conversations*,* but it’s never the same as having that face to face presence within the practice.” (Interview 10*,* GP)*.

The ANs reported that the first few months of the pandemic presented a major challenge in receiving patient referrals due to the delays in GPs seeing patients in-person. This led to reduced referral rates during this time, compared to before the pandemic. One GP reported that that this was down to their practice ceasing health assessments for identifying alcohol and other problems with new patients, and the difficulty in determining the extent of a patient’s alcohol problem in non-face to face appointments:

*I think it’s* [COVID-19 pandemic] *massively impacted on the volume* [of referrals] *because quite often we would pick things up by seeing patients… a skin lesion looked at*,* and you notice their breath smells of alcohol and then you go into that conversation. So*,* it’s that whole kind of opportunistic part of it is completely lacking and it’s not something you can do over the phone.*

However, PCANOS did see an increase in referrals during this initial period for patients who did not meet their eligibility criteria, but who still required support for an alcohol problem– these referrals came from the mainstream alcohol services, the ADRS, which were reportedly overwhelmed with patients at this time.

Some patients reported increased difficulty to reduce their alcohol consumption during this time for many reasons including being unable to engage in certain coping strategies (e.g. going outside for exercise) and boredom due to a lack of things to do. The PCANOS team quickly adapted to not being able to visit patients in the first two months of the pandemic by using video and telephone calls:

*“… the lockdown had hit and he* [AN] *could’nae* [couldn’t] *come out anymore. I think he was only out once or twice and then it got really bad with the COVID… But he did phone and that and I think*,* see if it wasn’t for him* [AN] *like he was really helpful*,* like with services and that. But see because of all the COVID stuff and that*,* the groups he was talking about*,* they would have been brilliant… the walking group and I think… it was a few other groups. But medications as well*,* he was really good with medications and I think he was on the phone to my doctor and that just making sure.” (Interview 22*,* Patient)*.

### Comparison of PCANOS to other mainstream alcohol services

Participants were asked to reflect on how their experiences of PCANOS compared to those of the mainstream alcohol services in Glasgow, the ADRS. The ANs reported that they had a smaller patient caseload than their colleagues in the ADRS, and that this allowed them to respond more quickly to patients after a referral, providing an opportunity to engage with patients while they were more motivated to receive support. The smaller caseload also allowed them to spend more time engaging with patients and offer flexibility to patients. One patient who was towards the end of his 13-week period with the PCANOS team, and who had attempted to contact the ADRS, described his frustration with this experience and their inability to be flexible to his situation:

*“…I’ve only spoke to her* [someone from the mainstream alcohol service] *once and it was somebody else that contacted me before her and then there was a new woman I spoke to her for an hour one day… she said to me she can’t commit for me*,* to drop my case because I work in* [a supermarket] *and I work… all different shifts*,* so I can only see three weeks [ahead]… they have basically told me if I cannot give them a Wednesday at 1:00 for the foreseeable future*,* they will give someone the space and slot.” (Interview 21*,* Patient)*.

Some staff participants believed that the combined alcohol and drug services model that the ADRS operated led to two specific issues that affected patient engagement with the ADRS. The first was a perception that drug problems were given a higher priority than alcohol problems within the ADRS, which they believed resulted in patients with alcohol problems being missed out or not receiving effective care. The second was a perception that there was patient-associated stigma with attending a service that also addresses drug problems:

*“They’re* [ADRS] *are a good service but for a lot of people*,* particularly people who have alcohol problems*,* they see that service as a drugs service*,* when they go up there in a room and see its full of people that are using drugs then they’re uncomfortable. Particularly I think some of the women that have alcohol problems don’t like sitting in that environment.” (Interview 13*,* GP)*.

The ANs and practice staff reported that the good communication between PCANOS and general practices was not replicated with the ADRS. One practice staff member believed that the ADRS only communicated with GPs, and that this was a missed opportunity to increase communication with the entire general practice:

*“I think a lot of outside services don’t realise the role of the wider practice staff as well. The practice nurse is a fount of knowledge as well and so is our links worker. I know not every practice works like that*,* but there are more practices beginning to and I think a lot of services don’t tend to recognise that. They just think that the GP is the only person who they can talk to*,* when actually sometimes*,* if you talk to someone else*,* maybe the nurse or links worker*,* something can get done maybe quicker than having to wait for a GP who is already in surgery dealing with their surgery list*,* house calls…” (Interview 8*,* Practice Manager)*.

## Discussion

This study explored the views and experiences of stakeholders relating to PCANOS, an assertive outreach service that supported people with alcohol problems who were not engaging with mainstream services. To our knowledge, this is the first study to report on a model of alcohol assertive outreach that spanned both primary and secondary care and involved close, collaborative working between specialist addiction nurses and general practice staff, and thus the PCANOS model can be regarded as unique.

Though this was a small study, overall, our findings show the potential of PCANOS to engage with a specific patient group who needed specialist alcohol support, i.e. people living in the most deprived communities of Glasgow whose health was already negatively affected by their alcohol consumption, and who were not engaging with mainstream community services. This group is known to experience significant barriers to engaging with services [4, 5], and experience high levels of alcohol-related harms including being hospitalised and death [1, 31]. Thus, the study findings suggest PCANOS played an important role by engaging with this group of patients and providing much needed specialist support that may have not been received otherwise.

Although the word ‘assertive’ was not used by participants when referring to PCANOS, the key elements of the model highlighted in our findings align to those identified in the literature as common elements of assertive outreach [21, 32]. The PCANOS team provided outreach, i.e. they took the service to patients, mostly in their homes, and were assertive in that they made persistent and repeated attempts to contact referred patients, and engaged with them frequently for a 13-week period. The ANs provided person-centred care which involved being responsive and listening to the needs of patients, offering them flexibility, and building trustful relationships with them. The ANs’ specialist and wide-ranging skills and their collaborative working with general practice teams ensured a multi-disciplinary way of working to provide holistic and co-ordinated patient care.

We found that patients strongly supported PCANOS, as they reported benefits of engaging with the service including a reduction in their alcohol consumption and improvement in other aspects of their health and wellbeing, including diet, sleep and mental health. Our findings are comparable to findings from the evaluations of other types of alcohol assertive outreach services in England, which targeted different patient groups, all of whom had little to no engagement with mainstream services [33–35]. We also identified that there was strong support for PCANOS from the general practice staff, based on their experiences of good communication and increased support in caring for patients. This GP and patient support for PCANOS was also reported in a separate evaluation of PCANOS carried out by the Glasgow ADRS in 2022 [36]. That evaluation also examined the referral data for PCANOS between October 2019 and November 2021, and found that when comparing the three months before and three months after referral periods, there was a reduction of one GP contact, a 39% decrease in emergency departments attendances, and a 32% decrease in hospital admissions per patient [36]. While there were limitations to this evaluation, its findings combined with ours show the potential benefits that PCANOS might have, both to patients and healthcare services.

Another key finding from our study was that participants felt that the mainstream specialist alcohol services, the ADRS, did not provide the level of person-centred care necessary to engage and support the patient group that PCANOS supported due to having a higher caseload compared to PCANOS. This raises the question of whether patients who were discharged from PCANOS went on to successfully engage with the ADRS. We did not explore this in our study, but this would be useful to measure as part of future research. The issue of caseloads highlights the potential trade-offs that may have to be made when trying to engage with certain patient groups that need specialist alcohol support, i.e. smaller caseloads but potentially higher cost savings for services if these patients are successfully treated. For example, an evaluation of one assertive outreach service in England estimated a saving of £142,838 for engaging with 16 “high impact, change-resistant problem drinkers” [37]. We did not explore views on potential savings resulting from the operation of PCANOS, but it would be useful to explore this in future research.

The other issue that we identified with the ADRS was a concern that their combined focus of alcohol and drug treatment was perceived as a barrier to engagement with patients seen by PCANOS. This concern has also been expressed in a wider debate about alcohol treatment across Scotland, due to the latest evidence showing that an increased scrutiny of drug-related harms over recent years and subsequent focus on drug treatment has overshadowed alcohol treatment [4]. This is believed to be one of the main reasons for the 40% decline in the numbers of people accessing alcohol treatment in Scotland between 2013/14 and 2021/22 [38, 39]. There have been calls for more funding for services focused solely on alcohol to try to reduce the high levels of alcohol-related harms in the nation [38].

Our study also highlights the important role of general practice staff as part of the PCANOS model. They were crucial to identifying eligible patients, i.e. identifying the signs of alcohol dependence, and to raising awareness of PCANOS among patients and referring patients on. This highlights the continued need to provide training to general practice staff to support patients with alcohol problems, and for consistent funding of specialist services like PCANOS for patients to be referred to.

Given the potential of PCANOS highlighted by our study, there is value in further exploring this model of assertive outreach to measure its impact on patient outcomes and the wider economic and non-economic impacts on the healthcare system, including general practices. Unfortunately, PCANOS stopped operating in 2024, but there may be benefit in exploring refunding it in the future and conducting more robust research to understand its potential effectiveness compared to other alcohol treatment services. There may also be value in exploring if a model like PCANOS could benefit other population groups such as those living in rural areas who often face barriers to accessing alcohol treatment due to having to travel to large centres for treatment [40].

### Strengths and limitations

This is the first study from Scotland that qualitatively explored an alcohol assertive outreach service for people from the most deprived communities, who have an alcohol problem and who did not engage with mainstream services. Our findings add to the small body of published literature on this topic, and build understanding of factors which facilitate engagement with specialist alcohol treatment and care, highlighted as an area for alcohol research focus in Scotland [41].

There were several limitations to our study. The number of participants was small and it possible that we may have found more varied findings with a larger sample. There may have been sampling bias, for example, we did not manage to recruit patients who did not engage with PCANOS, or who may have left the service before being discharged. Their views may have differed from the views of the patients we recruited. Similarly, we did not recruit general practices that refused to sign up to refer patients to PCANOS, and their views might have differed from those of the general practice staff that we recruited.

## Conclusion

Findings from this study suggest that PCANOS had the potential to engage patients from the most deprived communities in Glasgow, who were not engaging with mainstream alcohol services. The service contained key elements of an assertive outreach service, including that it was delivered mainly in patients’ homes and used a person-centred care approach to support patients. The combination of knowledge and skills from the specialist alcohol nurse team based in secondary care, with those of general practice teams based in primary care, led to multi-disciplinary and collaborative ways of working, to provide holistic and co-ordinated patient care. The potential benefits of assertive outreach services like PCANOS should be explored more robustly to understand their role within the wider healthcare system. Future research should explore the impact on patient outcomes and economic impact in the healthcare context. Sufficient and consistent funding is necessary to keep specialist services like PCANOS operating, in order to support general practices and the wider healthcare system.

## Electronic supplementary material

Below is the link to the electronic supplementary material.

Supplementary Material 1

Supplementary Material 2

Supplementary Material 3

Supplementary Material 4

Supplementary Material 5

## Data Availability

The datasets generated and analysed during the study are not publicly available as consent for sharing this data beyond the study was not obtained from participants.

## Declarations

### Supplementary Information

The topic guides used in the interviews for the study are available as additional files.

Additional File 1– Interview guide Alcohol Nurse.

Additional File 2– Interview guide practice staff.

Additional File 3– Interview guide strategic staff.

Additional File 4– Interview guide service users.

Additional File 5– Summary of coding framework.

## Abbreviations

ADRS: Alcohol and Drugs Recovery Service
AN: Alcohol nurse
CAT: Community Addiction Team
GP: General practitioner
LW: Link worker
PCANOS: Primary Care Alcohol Nurse Outreach Service
