# Supplementary material for: Exploring views and experiences of a unique alcohol assertive outreach model, the primary care alcohol nurse outreach service (PCANOS): a qualitative study
Source: BMC Prim Care. 2025 Mar 3;26:61. doi: 10.1186/s12875-025-02755-8 (PMC11874102; doi:10.1186/s12875-025-02755-8)
Supplement: Supplementary file 1 — Supplementary Material 1 [file 12875_2025_2755_MOESM1_ESM.docx]

**Additional file 1:**

**Exploring the management of alcohol problems in Deep End practices in Scotland – interview guide for attached alcohol nurses (frontline staff)**

1. **Participant background**
   1. Please tell me a little about yourself in terms of your occupation and professional background.
   2. How did you come to work as an attached alcohol nurse (AAN) for this pilot? *Prompt: headhunted/applied. Ask participant to provide details about this process.*
   3. What were your duties as the AAN? *Prompt: day to day duties/responsibilities; activities carried out. List of activities carried out as part of the AAN pilot include (for each, ask them to provide details of what this entailed e.g. how often this was done):*

- *Physical health checks*
- *Child & Adult Support Protection risk assessments*
- *Mental health assessments*
- *Harm reduction*
- *Relapse prevention*
- *Dietary advice*
- *Joint care planning*
- *Initiation of interventions (using a motivational approach for individuals with co-created recovery strategies) – what type of interventions were offered?*
- *Home-supported detoxifications*
- *Referrals to alcohol and drug recovery services*
  1. Did your duties/responsibilities change over time of the 12-month pilot, and did this differ according to the GP practice? *If yes, ask what were the reasons for this, and how had they changed.*

1. **Preparing for and working with Deep End practices**
   1. How were practices chosen to be included in the AAN pilot?
   2. Were there any initial preparations or meetings that took place to plan for the pilot? Did this differ according to practice?
   3. Was there any opposition to the pilot running within the participating/selected practices? *If yes, ask who opposed and why.*
   4. Can you describe your experiences of working with the practices once the pilot started?

*Prompt: what went well?
Prompt: what problems were encountered?
Prompt: did this differ by practice?
Prompt: what would you do differently?*

1. **Service user involvement**
   1. Can you describe the types of service user/patients who engaged with the AAN pilot?

*Prompt: reasons for engaging?
Prompt: for an anonymised example of a patient who received help, who would otherwise not have received help?*

- 1. Can you describe the types of service user/patients who did not engage with the AAN pilot?

*Prompt: reasons for not engaging?
Prompt: for an anonymised example of a patient who the AAN pilot was unable to help, and reasons for this?*

- 1. What factors influenced the uptake of the AAN service?

*Prompt on service-level factors which helped or hindered uptake? E.g. partnership working, flexible working, referrals process, contacting patients, others?
Prompt on patient-level factors which helped or hindered uptake? E.g patient attitudes to seeing the AAN, how did these change over time?*

1. **Views about the pilot**
   1. How successful do you think the AAN pilot was in addressing the needs of patients in the practice?

*Prompt: reasons why
Prompt: what they view as ‘success’*

- 1. What were the advantages/disadvantages to being located in the Deep End practices? Would these be different for practices not located in deprived areas?
  2. How does the treatment/support of the AAN pilot compare to that offered in the community? What are the advantages and disadvantages of treatment offered in the community?

1. The AAN is being rolled out across Glasgow again – what are your opinions about this? *If AAN is involved in current rollout, ask if they would do anything differently this time around in terms of engaging with practices and service users.*
2. Is there is anything about the pilot you want to talk about that we have not mentioned?
