# Supplementary material for: Exploring views and experiences of a unique alcohol assertive outreach model, the primary care alcohol nurse outreach service (PCANOS): a qualitative study
Source: BMC Prim Care. 2025 Mar 3;26:61. doi: 10.1186/s12875-025-02755-8 (PMC11874102; doi:10.1186/s12875-025-02755-8)
Supplement: Supplementary file 2 — Supplementary Material 2 [file 12875_2025_2755_MOESM2_ESM.docx]

**Additional file 2:**

**Exploring the management of alcohol problems in Deep End practices in Scotland – interview guide for practice staff (frontline staff)**

1. **Participant background**
   1. Please tell me a little about yourself in terms of your occupation and professional background.
   2. What is your role in relation to delivering services to or treating people with alcohol problems?

*Prompt: diagnosing alcohol problems; prescribing medication to help with alcohol problem, etc.*

- 1. What was your role in relation to the Deep End service (either the AAN pilot or PCANOS).

*Prompt: overall and day to day duties/responsibilities; referring patients to alcohol nurse; having meetings with alcohol nurse, etc.*

- 1. **For interviewees involved in the AAN** **only**: Did your duties/responsibilities change over time of the 12-month pilot?

*If yes, ask what were the reasons for this, and how had they changed.*

1. **Management of alcohol problems within the Deep End general practices**
   1. How does your practice currently support people with alcohol problems?

*Prompt: both in terms of the Deep End service but outside of this as well.*

- 1. What are the key challenges within the practice for delivering care to patients with alcohol problems?

*Prompt: challenges for practices and staff
Prompt: challenges for patients*

- 1. What would an ideal service that supports people with alcohol problems in the practice look like?

1. **The Attached Alcohol Nurse Pilot and/or PCANOS service**
   1. One attempt to improve care for people with alcohol problems was the Attached Alcohol Nurse or the newer PCANOS service – can you tell me how this was implemented in your practice?

*Prompt: How and why the practice came to be involved?
Prompt: What was involved in the preparations?*

*Prompt: What was/is involved in the day-to-day processes?
Prompt: What key challenges were encountered?
Prompt: What helped?*

- 1. Can you describe your experiences of working with the AAN pilot or PCANOS service?

*Prompt: what went well?
Prompt: what problems were encountered?
Prompt: what would you do differently?*

- 1. Can you describe the types of service user/patients who engaged with the AAN pilot and/or PCANOS service?

*Prompt: reasons for engaging?
Prompt: for an anonymised example of a patient who received help, who would otherwise not have received help?*

- 1. Can you describe the types of service user/patients who did not engage with the AAN pilot and/or PCANOS service?

*Prompt: reasons for not engaging?
Prompt: for an anonymised example of a patient who the AAN pilot was unable to help, and reasons for this?*

- 1. What factors influenced the uptake of the AAN pilot or PCANOS service within your practice?

*Prompt on service-level factors which helped or hindered uptake? E.g. partnership working, flexible working, referrals process, contacting patients, others?
Prompt on patient-level factors which helped or hindered uptake? E.g patient attitudes to seeing the AAN*

- 1. How successful do you think the AAN pilot or PCANOS service was/is in addressing the needs of patients in your practice?

*Prompt: reasons why
Prompt: what they view as ‘success’*

- 1. What were the advantages/disadvantages to having an alcohol nurse attached located in your practice?
  2. What do you think about the future or sustainability of the PCANOS service?

*If the practice is involved in the current rollout, ask why they have chosen to be involved again, if there are any differences in terms of planning or approach this time around compared to the initial pilot.*

*If the practice is not involved in the current rollout, ask why and if they would be willing to have an alcohol nurse located in their practice if they were asked.*

1. **Community-based alcohol services**
   1. Are there any alcohol services that are available to your practice patients in the local community?
   2. What are the key challenges for these community-based services in delivering care to patients with alcohol problems?

*Prompt: challenges for services
Prompt: challenges for patients*

- 1. What would an ideal community-based alcohol service look like?
  2. How does treatment or support delivered by the alcohol nurse differ from that received within the community alcohol treatment services?
     *Prompt on advantages/disadvantages of treatment offered in the community*
