# Supplementary material for: Exploring views and experiences of a unique alcohol assertive outreach model, the primary care alcohol nurse outreach service (PCANOS): a qualitative study
Source: BMC Prim Care. 2025 Mar 3;26:61. doi: 10.1186/s12875-025-02755-8 (PMC11874102; doi:10.1186/s12875-025-02755-8)
Supplement: Supplementary file 3 — Supplementary Material 3 [file 12875_2025_2755_MOESM3_ESM.docx]

**Additional file 3:**

**Exploring the management of alcohol problems in Deep End practices in Scotland – interview guide for strategic staff**

1. **Participant background**
   1. Please tell me a little about yourself in terms of your occupation and professional background.
   2. What is your role in relation to delivering services to people with alcohol problems?
   3. What was your role in relation to the AAN pilot? *Prompt: overall and day to day duties/responsibilities.*
   4. Did your duties/responsibilities change over time of the 12-month pilot? *If yes, ask what were the reasons for this, and how had they changed.*
2. **Management of alcohol problems in primary care**
   1. What services are currently available within the Deep End and other GP practices in your area to help people with alcohol problems?
   2. What are the key challenges for these practices in delivering care to patients with alcohol problems?

*Prompt: challenges for practices
Prompt: challenges for patients*

- 1. What would an ideal service in primary care look like?

1. **The Attached Alcohol Nurse**
   1. One attempt to improve care for people with alcohol problems was the Attached Alcohol Nurse – can you tell me how that worked?

*Prompt: What worked well/less well?
Prompt: How well did it engage with patients?
Prompt: What type of patients engaged with the nurse?*

- 1. How well did the roll out of the pilot go?

*Prompt: what went well?
Prompt: what problems were encountered?
Prompt: what would you do differently?*

- 1. How successful do you think the AAN pilot was in addressing the needs of patients in primary care?

*Prompt: reasons why
Prompt: what they view as ‘success’*

- 1. Why was the AAN role initially stopped?
  2. What are the reasons for rolling out with AAN again? How does this current roll out differ from the initial pilot?

1. **Community-based alcohol services**
   1. What alcohol services are available to patients outside of primary care and within the community?
   2. What are the key challenges for these community-based services in delivering care to patients with alcohol problems?

*Prompt: challenges for services
Prompt: challenges for patients*

- 1. What would an ideal community-based alcohol service look like?
  2. How does treatment or support delivered by the AAN differ from that received within the community alcohol treatment services?
     *Prompt on advantages/disadvantages*
