# Supplementary material for: Exploring views and experiences of a unique alcohol assertive outreach model, the primary care alcohol nurse outreach service (PCANOS): a qualitative study
Source: BMC Prim Care. 2025 Mar 3;26:61. doi: 10.1186/s12875-025-02755-8 (PMC11874102; doi:10.1186/s12875-025-02755-8)
Supplement: Supplementary file 4 — Supplementary Material 4 [file 12875_2025_2755_MOESM4_ESM.docx]

**Additional file 4**

**Exploring the management of alcohol problems in Deep End practices in Scotland – interview guide for service users**

1. **Participant background**
   1. How are you feeling today? Is this a busy day for you? Have you got a lot planned for today? Thanks for giving us some of your time, etc.
   2. Can you tell me a little bit about yourself? *Prompt for demographic info: age, marital status, children, occupation, living situation*
   3. Can you describe your story in relation to your alcohol problem? *Prompt: when they started drinking and why, what issues have they experienced due to their drinking?*
   4. Did/does your alcohol problem have an impact on your health/wellbeing/family life/work life, etc? *Prompt: find out more about the specific impacts*
2. **Experiences with primary care/the general practice**
   1. Can you tell me about how you came to be in contact with [name of alcohol nurse]/PCANOS?

*Prompt: GP/practice staff put them in touch/they were concerned about their alcohol*  *problem or its impact on their health/wellbeing/family life, etc.*

*Prompt: How were they referred; How they felt about seeing the nurse; where did they meet; what was discussed during the meetings?*

*Prompt for what was discussed at the meetings: Physical health checks, Child & Adult Support Protection risk assessments, Mental health assessments, Harm reduction, Relapse prevention, Dietary advice, Joint care planning, Initiation of interventions (using a motivational approach for individuals with co-created recovery strategies), Home-supported detoxifications, Referrals to alcohol and drug recovery services.*

- 1. What did you find most helpful about seeing the alcohol nurse? *Prompt: reasons why*
  2. What did you find unhelpful? *Prompt: reasons why*
  3. What do you feel could have improved your experience with the alcohol nurse?
  4. Did you ever discuss your alcohol problem/drinking with your GP or GP staff or did they ever discuss your alcohol problem/drinking habits with you?

*Prompt: if yes, then why? Who helped you; what help was offered; what has been*  *most helpful and why; what has not been helpful/made it difficult for you to get help*  *and why;*

*If no, then why and would you have wanted them to discuss this with you?*

- 1. What should GPs or practices be doing better to help people with alcohol problems?

1. **Experiences with community addiction alcohol teams**
   1. Do you have any experience of receiving help for your alcohol problems from services provided by the community alcohol team or groups such as Alcoholics Anonymous? **OR** Has the alcohol nurse mentioned referring you to other community services once you have been discharged from PCANOS?

*Prompt: What type of treatment or support did they receive and how did it compare to the PCNAOS service OR how do they feel about going to another service after PCANOS?*

- 1. **If participant has experience of other community services**: What alcohol services provided in the community have been most helpful to you? *Prompt: which specific service provider are they talking about; reasons why helpful*
  2. **If participant has experience of other community services:** What alcohol services in the community have been least helpful?
     *Prompt: which specific service provider are they talking about; reasons why unhelpful*
  3. **If participant has experience of other community services:** How did the treatment or support you received in the community alcohol services or groups compare with the treatment or support you received from your general practice or alcohol nurse/worker?
  4. What would an ideal service to help people with alcohol problems look like if you were setting it up (in your general practice?)

**Debrief**

- Thank participant for their time.
- Ask if they would be interested in being sent the findings of the study, and if yes, what would be the best way to send the findings to them.
- If participant has mentioned any issues with alcohol/health, please signpost them to the following:
  - - Alcohol problems: Alcoholics Anonymous (AA) - <https://www.alcoholics-anonymous.org.uk/>, 0800 9177 650, email: help@aamail.org
    - Mental health: Samaritans – 116 123, email: [jo@samaritans.org](mailto:jo@samaritans.org) or visit a branch to speak face-to-face AND/OR Breathing Space (Scotland) - 0800 83 85 87
