# Supplementary material for: Exploring views and experiences of a unique alcohol assertive outreach model, the primary care alcohol nurse outreach service (PCANOS): a qualitative study
Source: BMC Prim Care. 2025 Mar 3;26:61. doi: 10.1186/s12875-025-02755-8 (PMC11874102; doi:10.1186/s12875-025-02755-8)
Supplement: Supplementary file 5 — Supplementary Material 5 [file 12875_2025_2755_MOESM5_ESM.docx]

**Additional file 5:**

**Summary of coding framework**

| **Code** | **Description** |
| --- | --- |
| Participant background | *Patients*: age, gender, employment status, interests, health issues  *Staff*: professional background and role, role in relation to PCANOS |
| Relationship with alcohol and drinking | *Patients*: drinking history and behaviours; reasons for drinking; reasons for seeking help; engagement with GP/other services for drinking problem |
| Experiences of the PCANOS service | *Patients*: Referral process, personal goals; views about AN; treatment received; impact on drinking and health; coping mechanisms; challenges experienced  *Staff*: aim of the service; how it operates; who is involved; type of patients seen; what has worked well; challenges |
| Views about other alcohol services | *Patients*: experiences and views of engaging with other services  *Staff*: experiences and views of other alcohol services |
